# Supplementary material for: Description and Genome-Based Analysis of Vibrio chaetopteri sp. nov., a New Species of the Mediterranei Clade Isolated from a Marine Polychaete
Source: Microorganisms. 2025 Mar 11;13(3):638. doi: 10.3390/microorganisms13030638 (PMC11945961; doi:10.3390/microorganisms13030638)
Supplement: Supplementary file 1 [file microorganisms-13-00638-s001.zip › microorganisms-3408044-supplementary-proofdone.pdf]

Description and genome-based analysis of *Vibrio chaetopteri* sp. nov. a new species of the Mediterranei clade isolated from a marine polychaete.

Valeriya Kurilenko [1,*], Evgenia Bystritskaya [1], Nadezhda Otstavnykh [1], Peter Velansky [2], Darina Lichmanuk [1], Yulia Savicheva [1], Lyudmila Romanenko [1] and Marina Isaeva [1,*]

[1] G.B. Elyakov Pacific Institute of Bioorganic Chemistry, Far Eastern Branch, Russian Academy of Sciences, Prospect 100 Let Vladivostoku, 159, Vladivostok 690022, Russia; valerie@piboc.dvo.ru (V.K.); chernysheva.nadezhda@gmail.com (N.O.); ep.bystritskaya@yandex.ru (E.B.); lichmanyukdarina@gmail.com (D.L.); iu.savicheva0@yandex.ru (Y.S.); lro@piboc.dvo.ru (L.R.); issaeva@gmail.com (M.I.)

[2] A.V. Zhirmunsky National Scientific Center of Marine Biology, Far Eastern Branch, Russian Academy of Sciences, Palchevskogo Street 17, Vladivostok 690041, Russia; velansky.pv@gmail.com (P.V.)

* Correspondence: valerie@piboc.dvo.ru (V.K.); issaeva@gmail.com (M.I.); Tel.: +7-423-231-1168 (V.K.)

Supplementary Materials

Table S1. MLSA primers designed in this study for PCR-amplification of *ftsZ*, *gyrB*, *rpoA*, *gyrA*, and *mreB* gene fragments

Gene	Primer name	Sequence (5′→3′)	Length bp	GC-content %	T_m, °C	Amplicon size, bp
ftsZ	Vibrio_ftsZ_F	CCGATGATGGAAATGTCTGACG	22	50,0	56,6	709
	Vibrio_ftsZ_R	CTTCYTCHGCDCGRTCTTCRCC	22	59,8	60,2	
gyrB	Vibrio_ gyrB _F	GCDATGCARTGGAAYGAYGG	20	54,2	55,9	561
	Vibrio_gyrB _R	YTGRTTYTTACGRTTACGRCC	21	45,2	51,1	
rpoA	Vibrio_ rpoA _F	CYGARGGCAAAGAYGAAGTGTT	22	47,7	55,0	495
	Vibrio_ rpoA _R	CAGGACGYARTAGRATHGGATC	22	49,2	51,2	
gyrA	Vibrio_ gypA _F	GGTCGTGCHCTTCCDGATGT	20	58,3	54,9	458
	Vibrio_ gypA _R	GGRATRTTDGTYGCCATACC	20	49,2	49,8	
mreB	Vibrio_ mreB _F	ATGAAAGAYGGHGTDATYGC	20	43,3	49,3	752
	Vibrio_ mreB _R	GCMACACANGTYAGWGGVTC	20	55,8	50,1	

Tm – annealing temperature; Notations for degenerate positions: Y = C/T, R = A/G, W = A/T, M = A/C, V = G/C/A, H = A/C/T, D = A/G/T, N = A/T/G/C

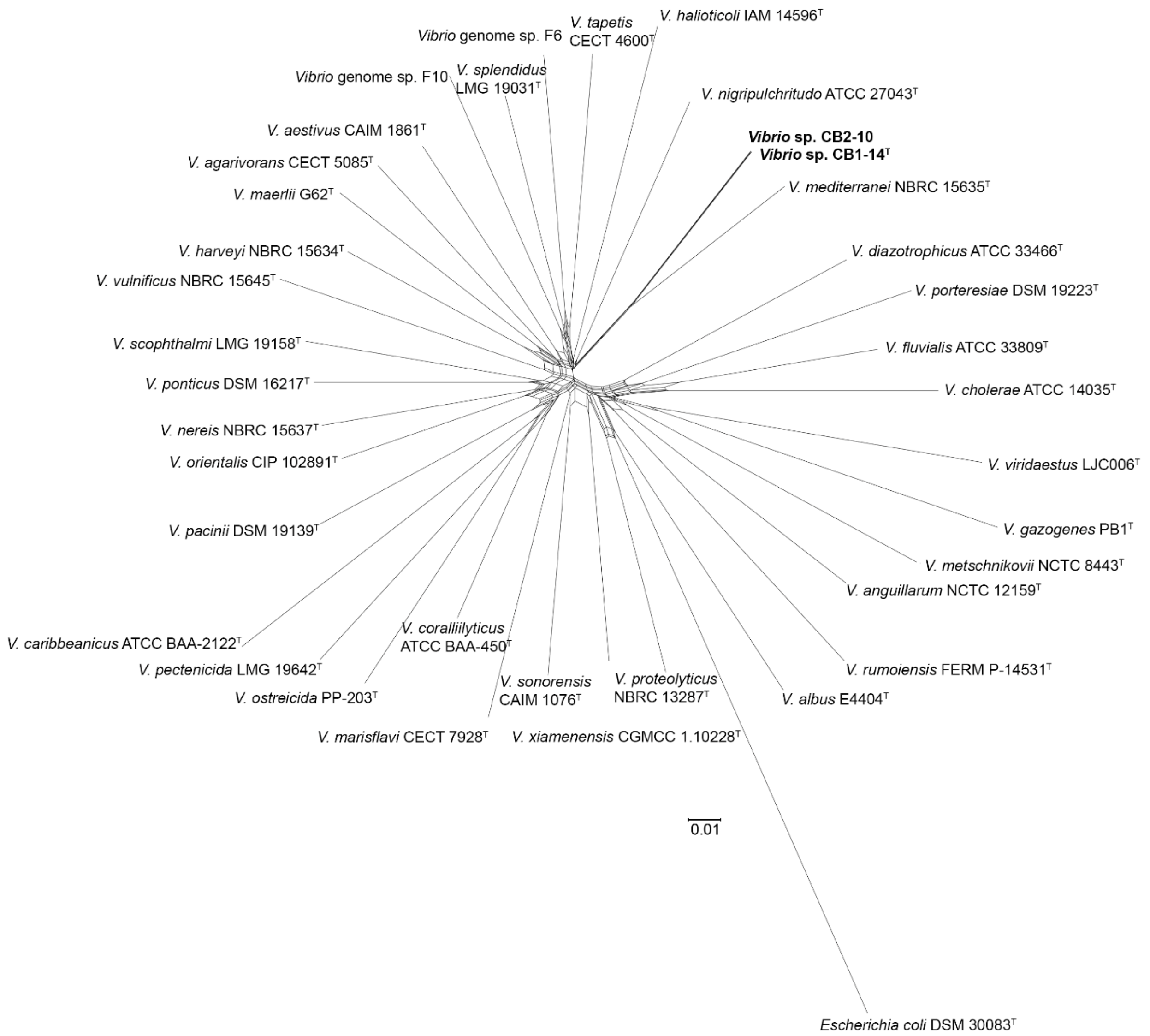

Figure S1. Eight-gene MLST neighbor-net phylogenetic network showing a phylogenetic position of new strains CB1-14^{T} and CB2-10 among 35 *Vibrio* type strains representing known *Vibrio* clades. *Escherichia coli* is used as an outgroup.

MLSA was conducted according to Jiang et al. (2022) [4], using sequences of eight housekeeping genes, *ftsZ*, *gapA*, *gyrB*, *mreB*, *pyrH*, *recA*, *rpoA*, and *topA*, which were retrieved from the GBK files of the *Vibrio* genomes using Geneious Pro software (version 4.8). All genes were aligned to corresponding ones of *V. cholerae* ATCC 14035^{T} (ASM62164v1) with gene positions 1-948, 1-960, 13-1650, 1-1038, 31-663, 1-906, 1-990, and 1-2073 for the *ftsZ*, *gapA*, *gyrB*, *mreB*, *pyrH*, *recA*, *rpoA*, and *topA* genes, respectively. Split decomposition analysis was performed for the concatenated genes using SplitsTree version 4.14.6 with a neighbor net drawing and a Jukes–Cantor correction [28].

Accession numbers for used genome sequences are following: GCF_001591125.1 (*V. mediterranei* NBRC 15635^{T}), GCF_038452265.1 (*V. diazotrophicus* ATCC 33466^{T}), GCF_024347055.1 (*V. porteresiae* DSM 19223^{T}), GCF_001558415.2 (*V. fluvialis* ATCC 33809^{T}), GCF_000621645.1 (*V. cholerae* ATCC 14035^{T}), GCF_003856525.1 (*V. viridaestus* LJC006^{T}), GCF_023920225.1 (*V. gazogenes* PB1^{T}), GCF_900460295.1 (*V. metschnikovii* NCTC 8443^{T}), GCF_900452855.1 (*V. anguillarum* NCTC 12159^{T}), GCF_002218045.2 (*V. rumoiensis* FERM P-14531^{T}), GCF_003144035.1 (*V. albus* E4404^{T}), GCF_000467125.1 (*V. proteolyticus* NBRC 13287^{T}), GCF_900100015.1 (*V. xiamenensis* CGMCC 1.10228^{T}), GCF_001854765.1 (*V. sonorensis* CAIM 1076^{T}),

GCF_921294215.1 (*V. marisflavi* CECT 7928^{T}), GCF_000176135.1 (*V. coralliilyticus* ATCC BAA-450^{T}), GCF_013074385.2 (*V. ostreicida* PP-203^{T}), GCF_024347015.1 (*V. pectenicida* LMG 19642^{T}), GCF_000165125.1 (*V. caribbeanicus* ATCC BAA-2122^{T}), GCF_000711795.1 (*V. pacinii* DSM 19139^{T}), GCF_000176235.1 (*V. orientalis* CIP 102891T), GCF_001591105.1 (*V. nereis* NBRC 15637T), GCF_009938225.1 (*V. ponticus* DSM 16217T), GCF_000222585.1 (*V. scophthalmi* LMG 19158T), GCF_002224265.1 (*V. vulnificus* NBRC 15645T), GCF_001591145.1 (*V. harveyi* NBRC 15634T), GCF_003415655.1 (*V. maerlii* G62T), GCF_030409635.1 (*V. agarivorans* CECT 5085T), GCF_003263845.1 (*V. aestivus* CAIM 1861T), GCF_001691155.1 (*Vibrio genome* sp. F10), GCF_024347615.1 (*V. splendidus* LMG 19031T), GCF_005146325.1 (*Vibrio genome* sp. F6), GCF_900233005.1 (*V. tapetis* CECT 4600T), GCF_003568965.1 (*V. halioticoli* IAM 14596T), GCF_000222685.1 (*V. nigripulchritudo* ATCC 27043^{T}) and GCF_003697165.2 (*Escherichia coli* DSM 30083^{T}). All genome sequences were downloaded from the NCBI GenBank (accessed on 18 January 2025).

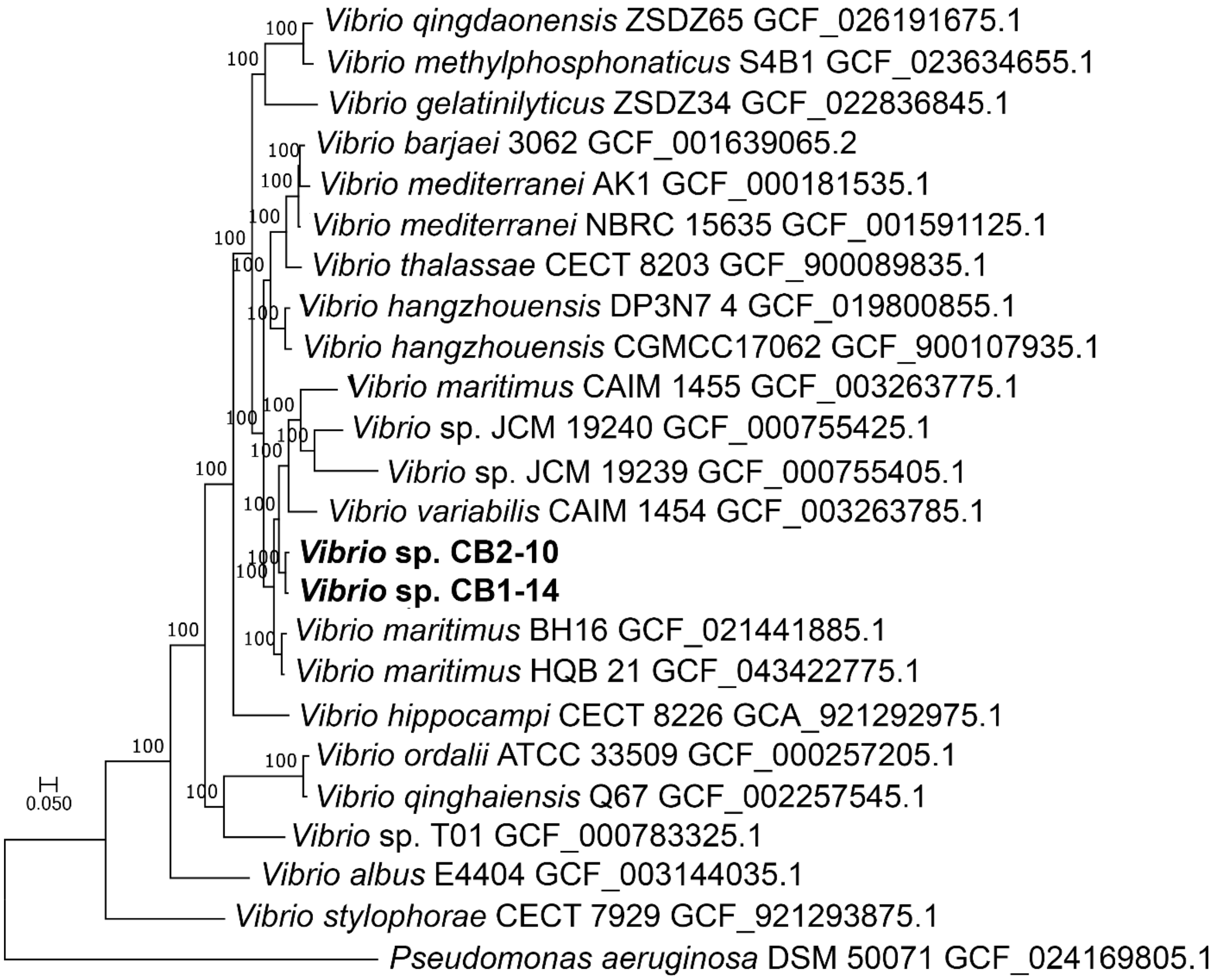

Figure S2. Position of new strains CB1-14^T (=KMM 8419^T) and CB2-10 (=KMM 8420), and related strains of the genus *Vibrio* designated to the Mediterranei clade based on concatenated sequences of 400 translated proteins. Bootstrap values are based on 100 replicates. Bar, 0.05 substitutions per amino acid position.

Figure S3: Two-dimensional thin-layer chromatograms of polar lipids of strains: (a) KMM 8419^{T}; (b) KMM 8420; (c) *V. thalassae* KCTC 32373^{T}.

Abbreviations: PE, phosphatidylethanoamine; LPE, lysophosphatidylethanolamine; PG, phosphatidylglycerol; DPG, diphosphatidylglycerol; APL, unidentified aminophospholipid; AL1, AL2, unidentified aminolipids; PL1, PL2, unidentified phospholipids; MHDG, monohexosyldiacylglycerol; HuDG, hexuronyldiacylglycerol; L1-L11, unidentified lipids; PA, phosphatidic acid.

a

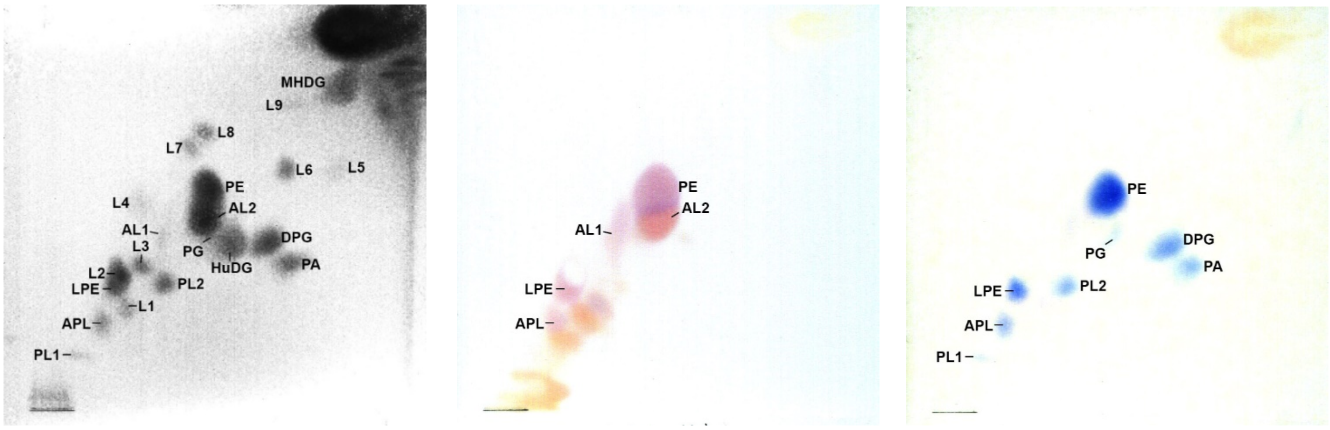

b

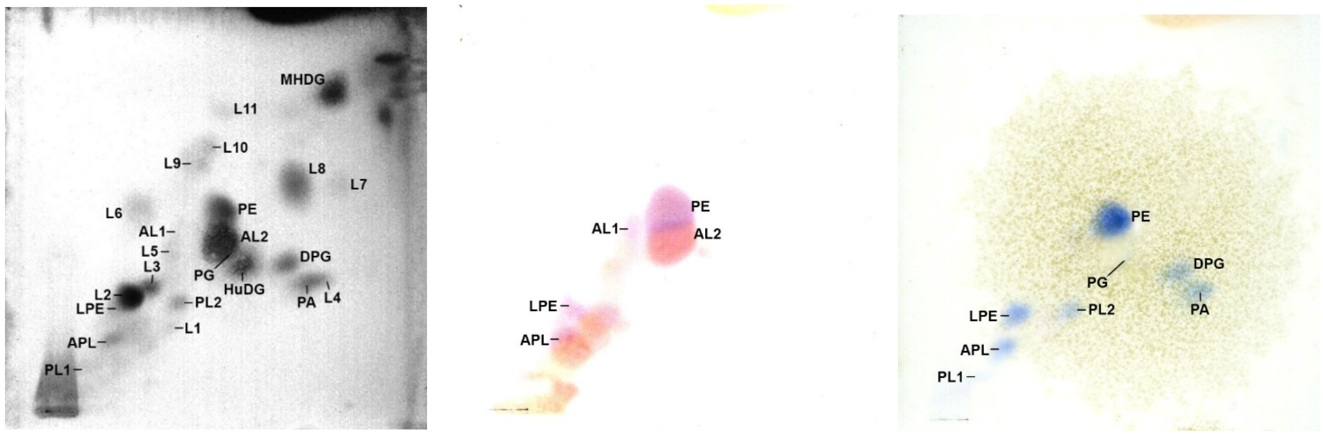

c

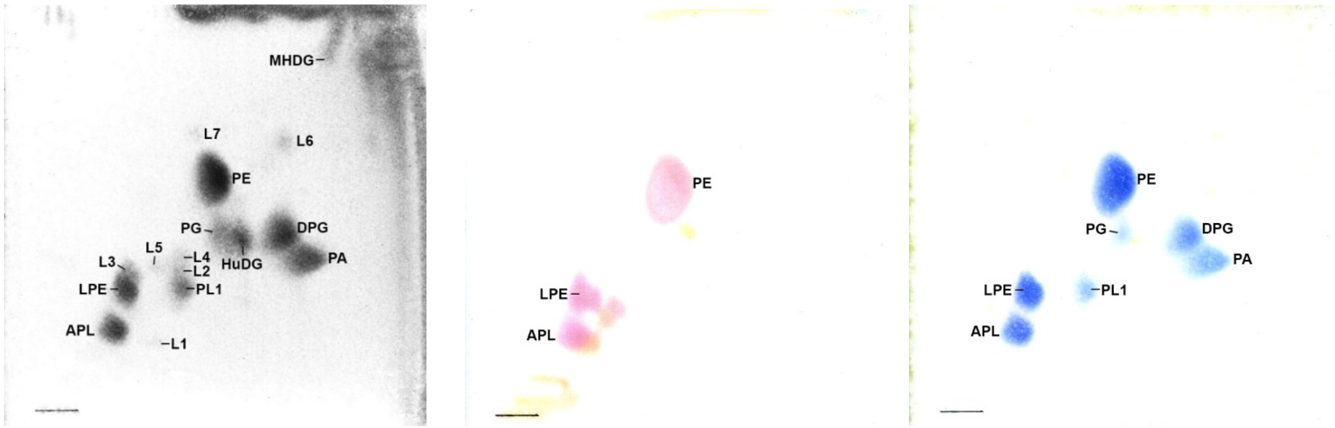

Table S4. Differential physiological characteristics of strains KMM 8419^T, KMM 8420 and *V. thalassae* KCTC 32373^T.

**Test**	**KMM 8419^T**	**KMM 8420**	***V. thalassae* KCTC 32373^T**
**API 20NE:**			
Nitrate reduction	+	+	+
Indole production	+	+	+
Glucose fermentation	-	-	-
Arginine dihydrolase	-	-	-
Urease	-	-	-
Hydrolysis (β- glucosidase) (esculin)	+	+	w
β-Galactosidase (PNPG)	+	+	+
Assimilation of:			
D-glucose	-	-	-
L-arabinose	-	-	-
D-mannose	-	-	-
D-mannitol	-	-	-
N-acetylglucosamine	-	-	-
D-Maltose	-	-	-
D-gluconate	-	-	-
Caprate	-	-	-
Adipate	-	-	-
L-malate	-	-	-
Citrate	-	-	-
Phenylacetate	-	-	-
**API 20E:**			
β-galactosidase (ONPG)	+	+	+
Arginine dihydrolase	-	-	+
Lysine decarboxylase	-	-	+
Ornithine decarboxylase	-	-	-
Citrate utilization	-	-	-
H_2S production under anaerobic conditions	-	-	-
Urease production under anaerobic conditions	-	-	-
Tryptophane deaminase	w	w	w
Indole production	+	+	+
Acetoin production	-	-	-
Oxidation of:			
D-glucose	+	+	+
D-mannitol	+	+	+
Inositol	-	w	-
D-sorbitol	-	-	-
L-rhamnose	w	w	-
D-sucrose	+	+	-
D-melibiose	-	w	-
Amygdalin	+	+	+
L-arabinose	-	-	-
Nitrate reduction	+	+	+
**API ZYM:**			
Alkaline phosphatase	+	+	+
Esterase (C 4)	+	+	+

Esterase Lipase (C 8)	+	+	+
Lipase (C 14)	+	w	-
Leucine arylamidase	+	+	+
Valine arylamidase	w	w	w
Cystine arylamidase	-	-	-
Trypsin	-	+	-
α-chymotrypsin	-	-	-
Acid phosphatase	+	+	+
Naphtol-AS-BI-phosphohydrolase	+	+	+
α-galactosidase	-	-	-
β-galactosidase	-	-	-
β-glucuronidase	-	-	-
α-glucosidase	w	+	+
β-glucosidase	-	-	-
N-acetyl-β-glucosaminidase	w	w	+
α-mannosidase	-	-	-
α-fucosidase	-	-	-
**Sensitivity to antibiotics:**			
Benzylpenicillin	+	+	-
Gentamicin<br>Neomycin<br>Chloramphenicol<br>Nalidixic acid<br>Tetracycline<br>Doxocycline<br>Rifampicin<br>Streptomycin	+	+	+
Vancomycin	+	-	-
Cephazolin	+	+	-
Polymyxin<br>Lincomycin<br>Kanamycin	-	-	-
Carbenicillin	-	-	+
Cephalexin	+	-	-
Ampicillin	-	+	+
Oxacillin	-	-	+

Symbol: (+) – positive, (-) – negative, (w) – week.
